# Pan-cancer analysis of PDZK1IP1 reveals its role in tumorigenesis and tumor immunity: focused validation in thyroid carcinoma

**DOI:** 10.1186/s41065-026-00662-1

**Published:** 2026-02-27

**Authors:** Chenchen Geng, Xiaoqian Gao, Yali Le, Shuzhen Zhu, Guanghui Zhao, Ping Zhang

**Affiliations:** 1 Department of Ultrasound, Qilu Hospital of Shandong University (Qingdao), Qingdao, Shandong Province China; 2 Health Management Center, Qilu Hospital of Shandong University (Qingdao), Qingdao, Shandong Province China; 3 Department of Clinical Laboratory, Qilu Hospital of Shandong University (Qingdao), Qingdao, Shandong Province China; 4 Department of Clinical Laboratory, Women and Children’s Hospital, Peking University People’s Hospital (Qingdao), Qingdao, Shandong Province China

**Keywords:** PDZK1IP1, Tumorigenesis, Immune microenvironment, Prognosis, Thyroid tumor

## Abstract

**Background:**

PDZK1IP1 is a membrane protein linked to inflammation and cancer, but its role in the tumor immune microenvironment is poorly understood. This study systematically investigated the oncogenic and immunological functions of PDZK1IP1 via a pan-cancer analysis, with experimental validation in thyroid carcinoma (THCA).

**Methods:**

We conducted a comprehensive bioinformatic analysis of PDZK1IP1 using publicly available platforms (TIMER, GEPIA, c-BioPortal, TISIDB, SangerBox) to examine its expression, diagnostic potential, prognostic relevance, and correlation with key immune features, including immune checkpoint genes, tumor mutational burden, neoantigen load, tumor stemness, and immune cell infiltration. Findings were validated in a THCA cohort using RT-PCR and immunohistochemistry.

**Results:**

PDZK1IP1 was overexpressed in multiple cancers, showed significant diagnostic potential, and was a context-dependent prognostic marker, correlating with poor relapse-free survival in THCA. Its expression was strongly associated with tumor mutational burden, neoantigen load, tumor stemness, and the infiltration of various immune cells (particularly neutrophils) across multiple cancers. These findings were corroborated in THCA, in which PDZK1IP1 expression correlated with the infiltration of five distinct immune cell types. Functional enrichment analysis confirmed an association with neutrophil activation pathways.

**Conclusions:**

Our study identified PDZK1IP1 with dual functions, acting as both an oncogene and a modulator of the tumor immune microenvironment. These findings highlight its potential as a prognostic biomarker and therapeutic target for immunotherapy, especially in THCA.

**Supplementary Information:**

The online version contains supplementary material available at 10.1186/s41065-026-00662-1.

## Background

PDZK1IP1-interacting protein 1 (PDZK1IP1), also known as MAP17, is a 17 kDa, nonglycosylated, membrane-associated protein primarily localized to the plasma membrane. It is characterized by a dual-pass transmembrane structure and a C-terminal PDZ-binding motif [1]. This motif facilitates interaction with PDZ domain-containing proteins, including PDZK1, the sodium-hydrogen antiporter 3 regulator (NHERF) family members 1–4, NaPiIIa, and the Na+/H+ exchanger 3 (NHE3) [2, 3]. Upregulated expression of PDZK1IP1 has been reported in chronic inflammatory diseases [4, 5]. For instance, José M et al. found that PDZK1IP1 expression directly regulates the activation of NFAT2 and IL-6 and induces the differentiation of monocytes into dendritic cells, implicating it in the inflammatory process. Furthermore, immunohistochemical analysis in Crohn’s disease and psoriasis has confirmed that sites of high PDZK1IP1 expression correlate with local inflammation, suggesting a role in regulating the immune microenvironment [6].

With the deepening of research, the role of PDZK1IP1 in tumorigenesis and progression has attracted more and more attention [7]. Some studies suggested it promoted tumor progression by regulating multiple pathways, such as by increasing reactive oxygen species (ROS) [8], activating the Notch pathway [9], and inhibiting the p38 pathway [10]. Conversely, other studies have demonstrated that PDZK1IP1 can also suppress tumor progression through several mechanisms. For instance, it can sequester the Smad4 protein to inhibit the TGF-β pathway, thereby impeding tumor cell growth [11]. In addition, increased PDZK1IP1 level was also related to poor prognosis and drug resistance [12–14]. However, existing research is largely preliminary and occasionally contradictory. Furthermore, most studies have focused on intrinsic tumor cell processes, leaving the relationship between PDZK1IP1 and the broader tumor immune microenvironment systematically unexplored.

In this study, we first employed bioinformatics methods to comprehensively analyze PDZK1IP1 in multiple tumors, examining its expression, prognostic relevance, and correlation with the tumor immune microenvironment. Among the various malignancies, thyroid carcinoma (THCA) emerged as a particularly compelling candidate for deeper investigation. THCA is a common endocrine malignancy with a global incidence that has risen significantly in recent years. Notably, it is far more prevalent in women than in men [15, 16]. THCA is classified into several main types based on its histopathological features and cell of origin: differentiated thyroid carcinoma, medullary thyroid carcinoma and undifferentiated thyroid carcinoma. Differentiated thyroid carcinoma contains papillary thyroid carcinoma, follicular thyroid carcinoma, and hürthle cell carcinoma [17].

Differentiated thyroid carcinoma is the most common category, accounting for over 90% of all cases, and generally carries an excellent prognosis [17]. The primary treatment approach involves surgical resection, often followed by radioactive iodine therapy and thyroid hormone suppression [18, 19]. However, for patients with advanced or undifferentiated cancers resistant to traditional treatments, immunotherapy has emerged as a promising strategy [20]. Therefore, identifying genes correlated with tumor immunity has become a research priority [21].

Given that our initial bioinformatic data indicated that PDZK1IP1 is not only highly overexpressed in THCA but also significantly correlates with its immune cell infiltration and poor relapse-free survival, we selected THCA as a primary model for experimental validation. Using RT-qPCR and immunohistochemistry, we aimed to confirm the clinical relevance of PDZK1IP1 in THCA, thereby providing a new perspective on its potential as a therapeutic target for immunotherapy.

## Methods

### Differential expression analysis of PDZK1IP1 in multiple tumors

The different mRNA expression level of PDZK1IP1 between tumors and the adjacent normal tissues was analyzed using the online platforms TIMER (https://cistrome.shinyapps.io/timer/) and GEPIA (http://gepia2.cancer-pku.cn/#analysis). We also analyzed mRNA expression levels of PDZK1IP1 in different tumor stages in the GEPIA database. The protein level of PDZK1IP1 between tumors and the adjacent normal tissues was analyzed through the Clinical Proteomic Tumor Analysis Consortium (CPTAC) data portal. All analyses were performed using the default parameters provided by each platform.

### Evaluation of the diagnostic value of PDZK1IP1 in pan-cancer

To assess the potential of PDZK1IP1 as a diagnostic biomarker for distinguishing tumor tissues from normal tissues, a pan-cancer Receiver Operating Characteristic (ROC) curve analysis was performed. Gene expression data (mRNA-seq) for various tumor types and their corresponding adjacent normal tissues were downloaded from the TCGA database (https://portal.gdc.cancer.gov).

For each cancer type with a sufficient number of available normal samples (*n* > 10), we conducted an ROC analysis using the pROC package in R (version 4.2.1). The expression level of PDZK1IP1 served as the predictor variable, and the sample type (Tumor vs. Normal) was the binary outcome variable. The Area Under the Curve (AUC) was calculated to quantify the diagnostic accuracy. An AUC value of 1.0 indicates a perfect diagnostic test, while a value of 0.5 suggests no diagnostic utility. The results were visualized by plotting the ROC curves for representative cancers and summarizing the AUC values across all analyzed cancer types.

### The prognosis was analyzed by Kaplan Meier plotter database

The prognostic significance of PDZK1IP1 mRNA expression was evaluated across 21 tumor types using the Kaplan Meier plotter database(http://kmplot.com/analysis/). The primary survival analysis indexes were overall survival (OS) and relapse-free survival (RFS). For each analysis, patients were stratified into high- and low-expression groups based on an automatically selected optimal cutoff value. Hazard ratios (HRs) with 95% confidence intervals (CIs) and log-rank *p* values were subsequently calculated.

### The relationship of PDZK1IP1 expression and Immune Checkpoint (ICP) Genes, Tumor Mutational Burden (TMB), neoantigen, ESTIMATE, and tumor stemness

Correlations between PDZK1IP1 expression and key tumor immune indicators were analyzed using TCGA pan-cancer data via the SangerBox online platform (http://www.sangerbox.com/tool) [22]. Specifically, we examined the association between PDZK1IP1 mRNA levels and the expression of a curated set of immune checkpoint (ICP) genes [23]. Tumor mutation burden (TMB) is a new marker, defined as the total number of non-synonymous mutations per megabase (mut/Mb) per coding area of a tumor genome, which can be used to predict the immunotherapy effect [24]. Neoantigen refers to tumor-specific DNA alterations that generate novel peptide sequences, usually without the average human genome [25]. Additionally, we analyzed the correlation of PDZK1IP1 expression with TMB and neoantigen.

At the same time, the Sangerbox platform can also estimate the proportion of immune components and matrix components in the tumor microenvironment (TME) and obtain three results- immune score, stromal score, and ESTIMATE score. The higher the immune or interstitial score, the higher the proportion of immune or interstitial in TME, indicating that the ratio of corresponding components in TME is more significant. The ESTIMATE score is the sum of the other two, representing the vast proportion of the two parts in TME [26]. The correlation between PDZK1IP1 expression and each of these three scores was then calculated across all cancer types.

Previous research confirmed the stemness index per TCGA tumor specimen and named it the “differentially methylated probes-based stemness index” (DMPsi) [27]. DMPsi is a quantitative metric, derived from a machine learning algorithm, that evaluates a tumor’s degree of “stemness” or dedifferentiation by comparing its DNA methylation profile to that of pluripotent stem cells. A higher DMPsi score signifies that the tumor is more epigenetically similar to an undifferentiated, primitive state. This index is clinically significant as it not only serves as a powerful measure of tumor malignancy and aggressiveness but has also been validated as a pan-cancer, independent predictor of poor prognosis, with higher DMPsi values strongly correlating with shorter patient survival. Furthermore, DMPsi reflects the activity of the internal cancer stem cell (CSC) population, which drives therapeutic resistance and relapse, giving it potential to guide future targeted therapies against stemness features and to reveal connections with the immunosuppressive TME. The correlation between PDZK1IP1 mRNA expression and the DMPsi score for each tumor sample was calculated using the Pearson coefficient. A *p* value less than 0.05 represented statistical significance.

### Exploring the relationship between PDZK1IP1 expression level and related immune cell infiltration in human tumors

Through the Sangerbox platform, the relationship between PDZK1IP1 expression in TME of 31 human tumors and six immune cells (B cells, CD4 + T cells, CD8 + T cells, neutrophils, macrophages, and dendritic cells) was analyzed by the TIMER method [28]. The *p* value < 0.05 was considered statistically significant.

### The functional enrichment analysis of the PDZK1IP1 related genes

A protein-protein interaction (PPI) network for PDZK1IP1 was constructed using the STRING database (version 12.0; https://string-db.org/). To perform a functional enrichment analysis, a composite gene list was created. This list included the top 100 genes co-expressed with PDZK1IP1 from the GEPIA database and the interacting proteins identified in the STRING network. This combined gene list was then subjected to Gene Ontology (GO) and Kyoto Encyclopedia of Genes and Genomes (KEGG) pathway analysis using the clusterProfiler package in R (version 4.2.1).

### The mRNA and protein expression of PDZK1IP1 in THCA samples

To explore the expression of PDZK1IP1 in THCA, we first collected 18 pairs of THCA and its matched paracancerous tissues. The patient cohort comprised fourteen pairs of papillary thyroid carcinoma (PTMC), two pairs of medullary carcinoma (MTC), one pair of follicular thyroid carcinoma, and one pair of undifferentiated carcinoma. We performed quantitative real-time polymerase chain reaction (RT-PCR) to detect the mRNA expression in the tumor and paracancerous tissues. Briefly, total RNA was extracted by TRIZOL (TAKALA, Dalian, China). One µg of total RNA was reverse transcribed into cDNA using the HiScript II Q RT SuperMix (Vazyme Biotech, Nanjing, China). RT-PCR was then performed using SYBR Green Real-Time PCR Master Mix (Vazyme Biotech, Nanjing, China). Ct values were normalized to GAPDH, and relative expression was calculated using the 2-^ΔΔCt^ method. Samples were analyzed in triplicate, and statistical analysis was performed using the t-test.

Immunohistochemistry (IHC) was performed to analyze PDZK1IP1 protein expression in six pairs of formalin-fixed, paraffin-embedded thyroid cancer and matched paracancerous sections. Tissues from surgery were stained with hematoxylin, and eosin standard immunohistochemical protocol was performed to assess protein expression of PDZK1IP1 (rabbit monoclonal, ab156014, Abcam, Cambridge, UK, 1:150 dilution). Images were analyzed using a quantitative digital image analysis system (Image-Pro Plus 6.0). The integrated optical density (IOD) and the corresponding staining area were measured, from which the average optical density (AOD) was calculated (IOD/AREA). The finally obtained data were analyzed by a paired t-test.

### Gene Set Enrichment Analysis (GSEA)

To further investigate the biological pathways and functions associated with PDZK1IP1, we performed GSEA. First, RNA-sequencing data and corresponding clinical information for the THCA cohort were downloaded from the TCGA database. The patient samples were stratified into high- and low-expression groups based on the median expression level of PDZK1IP1. Subsequently, Differential Expression Analysis (DEA) was conducted between the high- and low-PDZK1IP1 expression groups using the DESeq2 package in R (version 4.2.1). Genes with a |log2(Fold Change) | > 1 and an adjusted *p* value < 0.05 were identified as differentially expressed genes (DEGs).

For the GSEA, we utilized the entire set of genes ranked by their log2(Fold Change) values derived from the DEA. This pre-ranked list was then analyzed using the clusterProfiler package in R (version 4.2.1). Pathways with a False Discovery Rate (FDR) q value < 0.25 were considered significantly enriched, as per standard GSEA guidelines.

## Results

### PDZK1IP1 was differentially expressed in multiple tumors and normal tissues

**Fig. 1:**
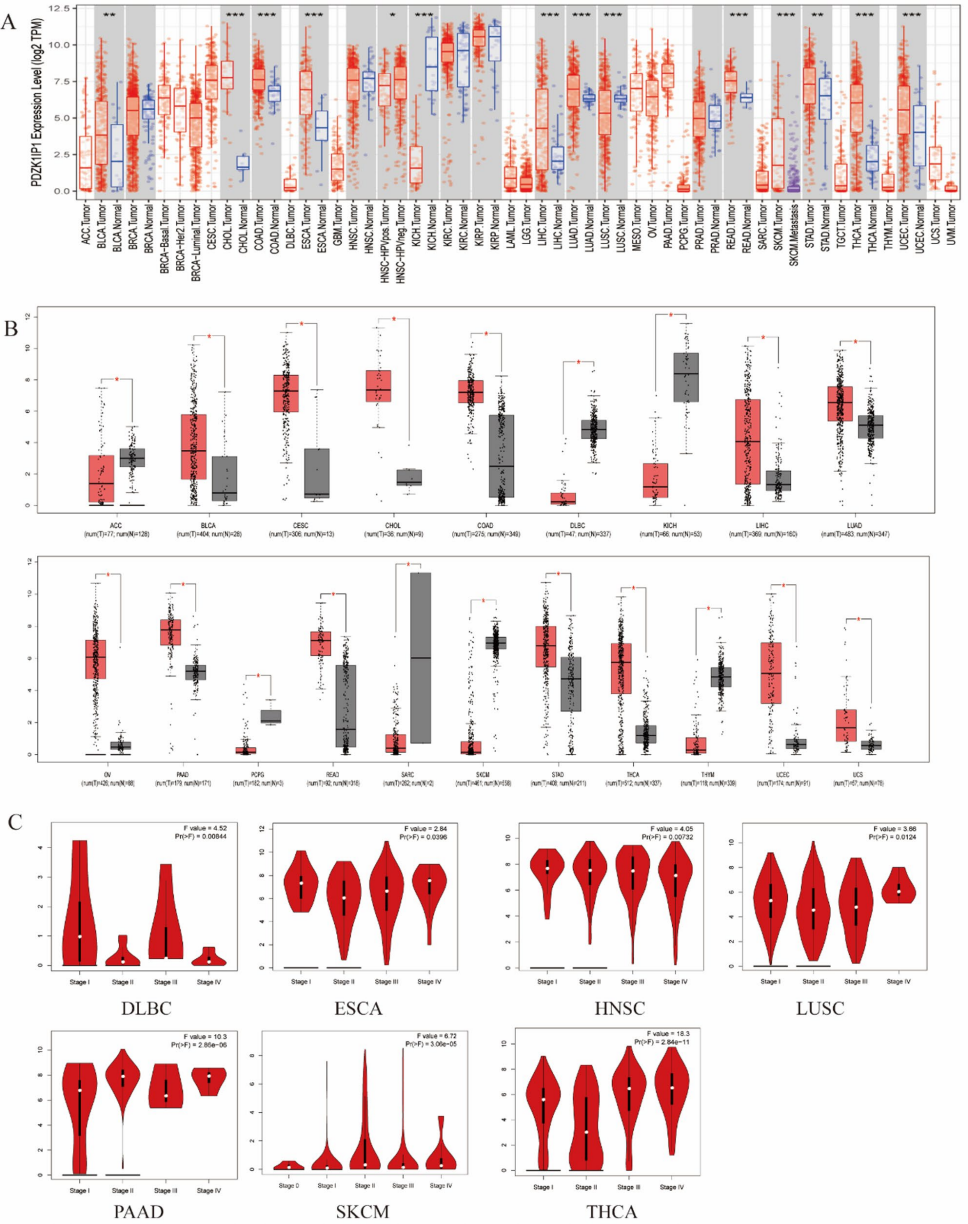
PDZK1IP1 expression level in multiple tumors. **A** PDZK1IP1 expression levels in different cancers from the TCGA database analyzed by the TIMER database. (*p < 0.05, **p < 0.01, ***p < 0.001). **B** PDZK1IP1 expression levels in different cancers and paired normal tissue in the GEPIA database. **C** PDZK1IP1 expression levels in different tumor stages in GEPIA database

In the Timer database, the mRNA expression level of PDZK1IP1 in some cancers, including THCA, CHOL (cholangiocarcinoma), COAD (colon adenocarcinoma), LIHC (liver hepatocellular carcinoma), LUAD (lung adenocarcinoma), READ (rectum adenocarcinoma), UCEC (uterine corpus endometrial carcinoma) was significantly higher than that in adjacent tissues. Conversely, its expression was significantly downregulated in KICH (Fig. 1A). These findings were largely corroborated by an independent analysis using the GEPIA database. The mRNA levels of PDZK1IP1 were markedly higher in multiple cancers, like THCA, COAD, LIHC, LUAD, PAAD (pancreatic adenocarcinoma), READ, UCEC (uterine corpus endometrial carcinoma), than those in normal adjacent tissues. Similarly, downregulation in KICH was also confirmed (Fig. 1B). Furthermore, we analyzed the expression level of PDZK1IP1 in different tumor stages and found significant differences in THCA and PAAD in GEPIA database (Fig. 1C). In the CPTAC database, which contained 13 tumors, we found that the protein levels of PDZK1IP1 in UCEC, LUAD, and PAAD were significantly higher than in adjacent tissues. However, the protein level in BRCA (breast invasive carcinoma) tissues was lower than in normal tissues (Supplementary Fig. 1). Taken together, these multi-database analyses consistently demonstrated that PDZK1IP1 was frequently overexpressed at both the mRNA and protein levels in multiple human cancers and that its expression correlated with more advanced tumor stages, particularly in THCA.

### Diagnostic value of PDZK1IP1 in pan-cancer

To assess the potential of PDZK1IP1 as a diagnostic biomarker, we performed a ROC curve analysis for various cancers. The results showed that PDZK1IP1 expression had significant diagnostic value in distinguishing tumor tissues from adjacent normal tissues. Notably, the AUC was high in several cancers, including KICH (AUC = 0.99), THCA (AUC = 0.85), READ (AUC = 0.84), and COAD (AUC = 0.77) (Supplementary Fig. 2). These findings suggested that PDZK1IP1 could serve as a promising diagnostic marker, particularly for kidney, thyroid, rectum and colon cancers.

### PDZK1IP1 was related to prognosis in multiple tumors

To evaluate the prognostic value of PDZK1IP1, we analyzed its association with patient survival using mRNA-seq data from the Kaplan-Meier plotter database. High PDZK1IP1 expression correlated with either poor or favorable outcomes depending on the cancer type. For example, high expression of PDZK1IP1 was associated with poor OS in some tumors, including PAAD (HR = 2.45, *p* = 0.0005), CESC (cervical squamous cell carcinoma and endocervical adenocarcinoma) (HR = 1.65, *p* = 0.033), LUSC (lung squamous cell carcinoma) (HR = 1.38, *p* = 0.04), THYM (thymoma) (HR = 5.88, *p* = 0.0043). However, high expression indicated better OS in HNSC (head and neck squamous cell carcinoma) (HR = 0.69, *p* = 0.0081) and KIRP (kidney renal papillary cell carcinoma) (HR = 0.53, *p* = 0.038) (Supplementary Fig. 3A-G), and better RFS in BRCA (HR = 1.7, *p* = 0.018), ESCA (esophageal carcinoma) (HR = 0.34, *p* = 0.025), KIRP (HR = 0.25, *p* = 0.015), LIHC (HR = 0.66, *p* = 0.012), STAD (stomach adenocarcinoma) (HR = 0.48, *p* = 0.035). Our finding in HNSC was consistent with a prior study that reported an association between high PDZK1IP1 expression and better OS in laryngeal cancer [29]. Notably, in THCA, high PDZK1IP1 expression was strongly associated with poor RFS (HR = 3.47, *p* = 0.00083) (Supplementary Fig. 3H-N).

### The expression of PDZK1IP1 was related to Immune Checkpoint (ICP) genes

Immune checkpoint (ICP) genes are associated with immune cell infiltration, and blocking immune checkpoint therapy with an anti-tumor immune response gene has become one of the immunotherapy strategies for cancer patients [30]. Through Supplementary Fig. 4, we found that PDZK1IP1 expression was significantly correlated with the majority of ICP genes in 18 tumor types, showing both positive and negative associations depending on the cancer and specific ICP gene. For the tumors with positive correlation, it indicated that PDZK1IP1 could be used as the immunotherapy target. For the tumors whose expression of PDZK1IP1 was negatively correlated with ICP genes, we comprehended this phenomenon as the higher the expression of PDZK1IP1, the worse the immunotherapy effect. Notably, in THCA, it was correlated with 34 ICP genes, which could provide a specific basis for selecting immunotherapy in the clinic.

### The expression of PDZK1IP1 was related to TMB, neoantigen, and ESTIMATE

**Fig. 2:**
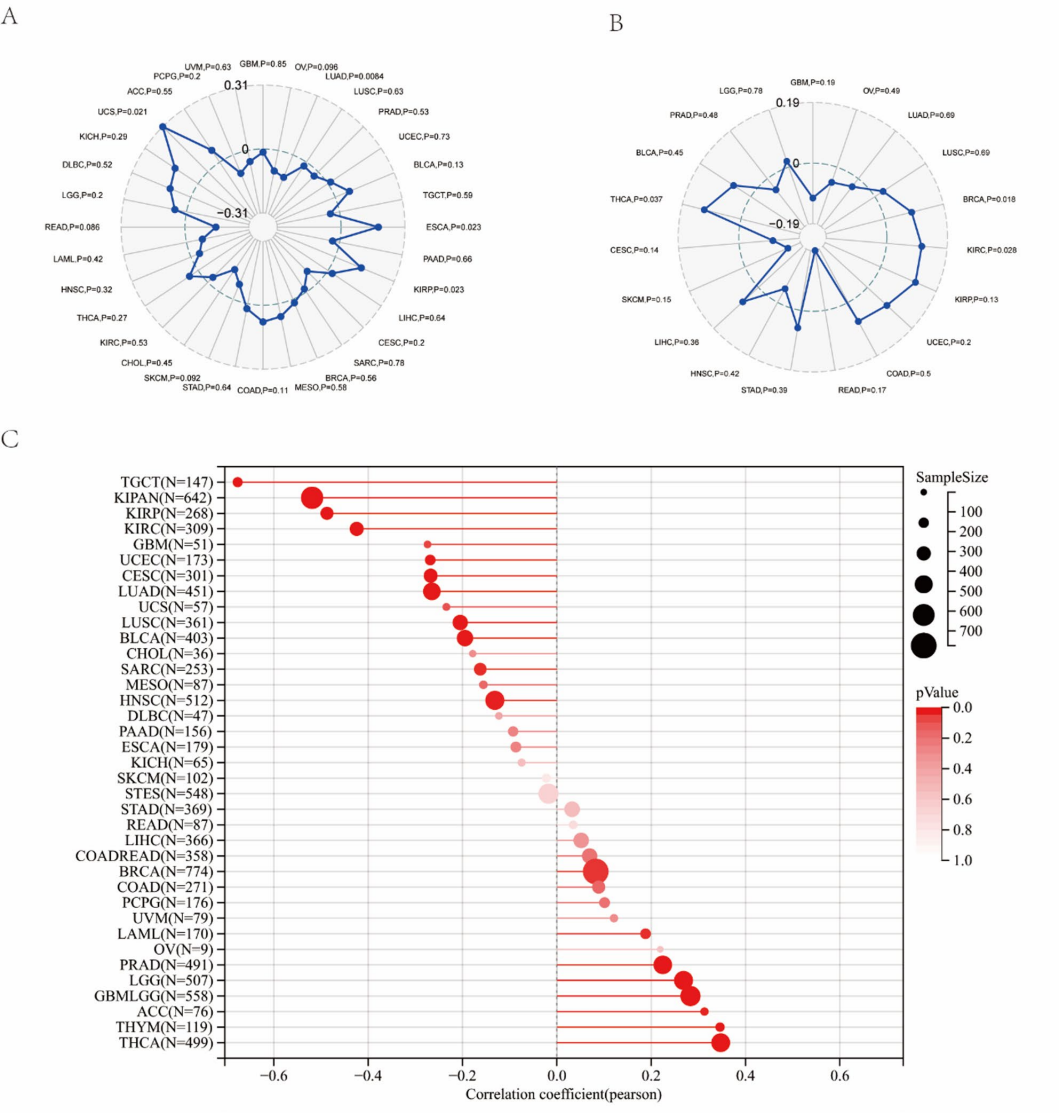
The relationship between PDZK1IP1 expression and TMB (**A**), neoantigen (**B**), and tumor stemness index (**C**) in human cancers. TMB, tumor mutational burden

To further investigate the role of PDZK1IP1 in tumor immunity, we analyzed its association with several key immune markers. First, we assessed the correlation between PDZK1IP1 and TMB. As a critical predictor of immunotherapy efficacy, a high TMB implies that a tumor carries more mutations. Our analysis revealed that its expression was positively correlated with UCS, KIRP, and ESCA and negatively correlated with LUAD (Fig. 2A). This suggested that in certain cancers, high PDZK1IP1 expression could be associated with greater tumor immunogenicity. To validate this, we directly analyzed its correlation with neoantigen and found that its expression was positively correlated with THCA, KIRC, BRCA (Fig. 2B). Next, we utilized the ESTIMATE algorithm to evaluate the relationship between PDZK1IP1 and the components of TME. The analysis revealed a broad pattern of association: PDZK1IP1 expression was significantly positively correlated with the total ESTIMATE score in as many as 21 cancer types (including OV, LUAD, LUSC, BRCA, etc.), and was negatively correlated in only SARC, MESO, and COAD (Supplementary Fig. 5). This widespread positive correlation strongly indicated that high PDZK1IP1 expression was generally associated with an “impure” TME, rich in immune and stromal cell infiltration. Taken together, these findings delineated a potential central role for PDZK1IP1 in tumor immunity. The expression of PDZK1IP1 was not only linked to the tumor’s capacity to generate targets for immune recognition (TMB and neoantigens) but was also closely associated with the actual infiltration of immune cells into the TME.

### The expression of PDZK1IP1 was related to tumor stemness index

We next evaluated the association between PDZK1IP1 expression and tumor stemness index across TCGA pan-cancer data in the Sangerbox database. It was found that PDZK1IP1 expression was significantly correlated with tumor stemness index in 19 cancer types. Specifically, a significant positive correlation was identified in 8 tumor types, including GBM, LGG, LAML, BRCA, PRAD, THYM, THCA, and ACC. Conversely, a significant negative correlation was found in 11 tumors, including CESC, LUAD, SARC, KIRP, KIPAN, UCEC, HNSC, KIRC, LUSC, TGCT, and BLCA. These results suggested that PDZK1IP1 may play roles in tumor progression, especially in the tumors with a significant positive correlation with tumor stemness index. As the PDZK1IP1 expression had positive correlation with THCA, we proposed that targeted PDZK1IP1 could inhibit THCA progression (Fig. 2C).

### The expression of PDZK1IP1 was related to immune cell infiltration in TME

**Fig. 3:**
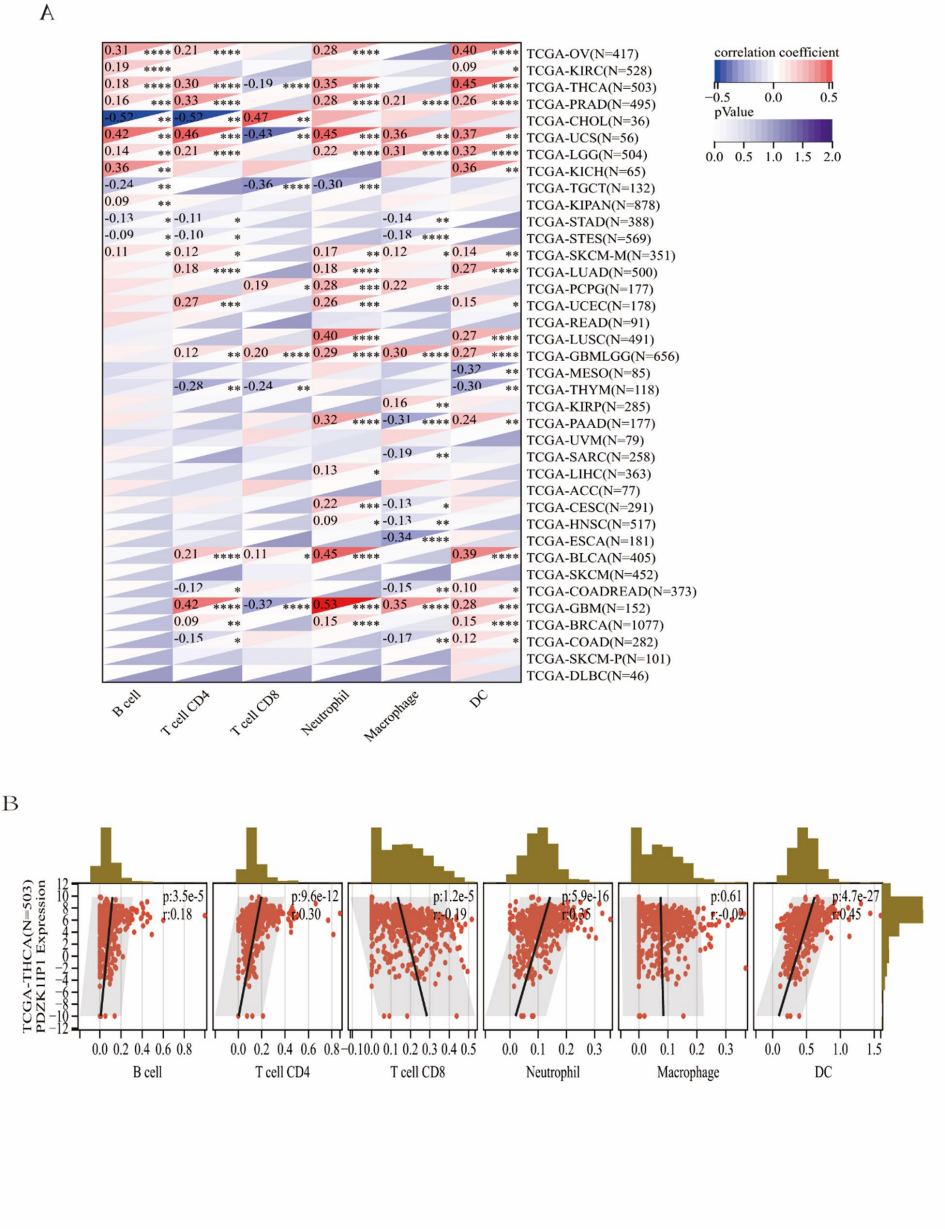
The relationship between PDZK1IP1 expression and infiltrating immune cells of human cancers and THCA. **A** In different cancer types, the relationship between PDZK1IP1 expression level and infiltrating levels of B cells, CD4 + T cells, CB8 + T cells, macrophages, neutrophils, dendritic cell. **B** In THCA, the relationship between PDZK1IP1 expression level and infiltrating levels of B cell lineages, CD4 + T cells, CB8 + T cells, neutrophils, macrophages, dendritic cell. *p < 0.05; **p < 0.01; ***p < 0.001

To investigate the role of PDZK1IP1 in TME, we analyzed the correlation between its expression and the infiltration levels of various immune cell types. Using the TIMER method in the Sangerbox database, we found that PDZK1IP1 expression was correlated with immune cell infiltration in 32 tumors (Fig. 3A). Among them, PDZK1IP1 expression was associated with B cells in 13 tumors, CD4 + T cells in 18 tumors, CD8 + T cells in 9 tumors, neutrophils in 20 tumors, macrophages in 17 tumors, and dendritic cells in 20 tumors. Notably, in our focus cancer type, THCA, PDZK1IP1 expression showed a significant and strong correlation with the infiltration of five distinct immune cell types: B cells, CD4 + T cells, CD8 + T cells, neutrophils, and dendritic cells (all *p* < 0.001) (Fig. 3B).

### PDZK1IP1 co-expression networks and pathway enrichment analysis correlated with immunity

**Fig. 4:**
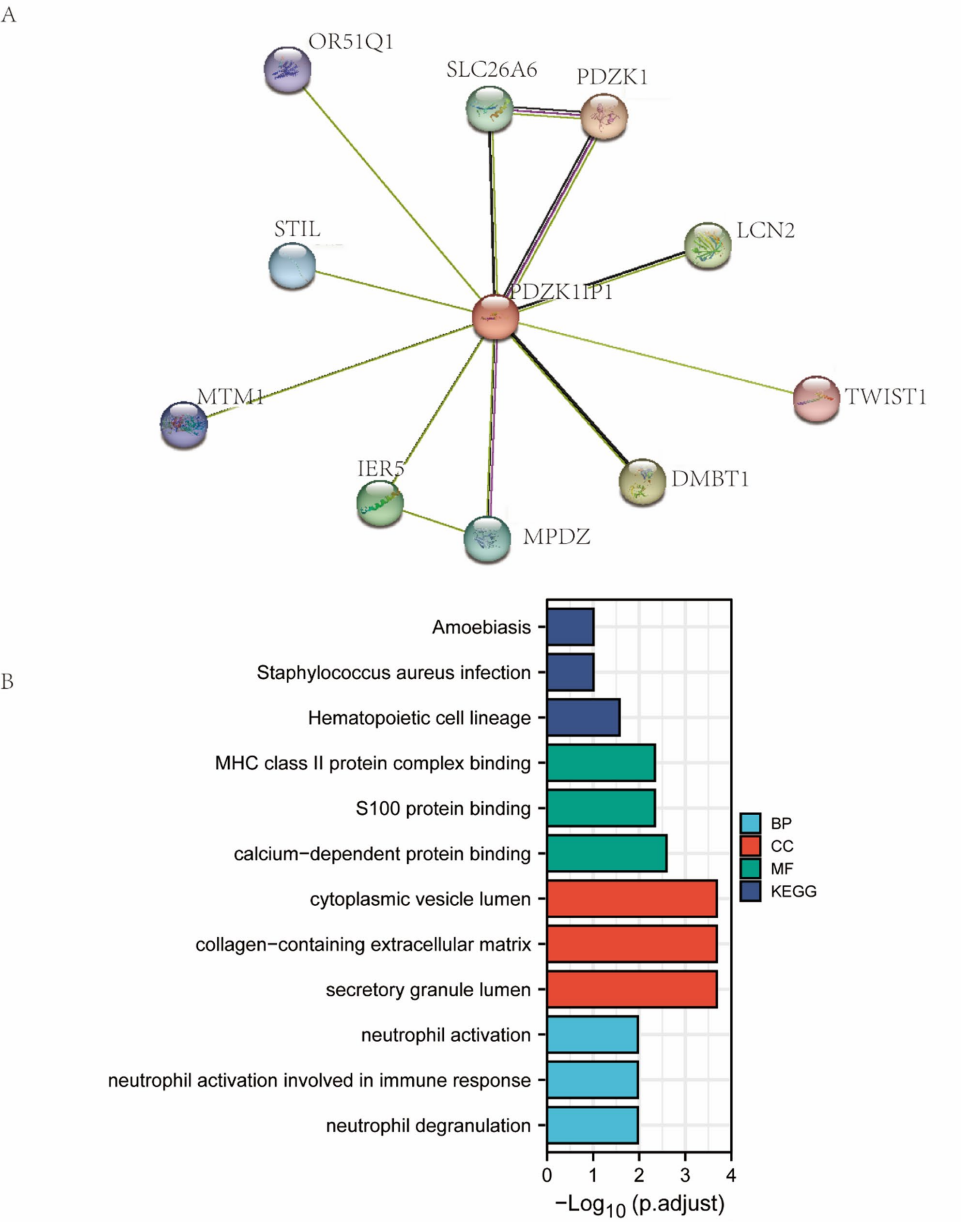
Enrichment analysis of PDZK1IP1 related proteins. **A** PDZK1IP1 related proteins on the String website. **B** GO and KEGG pathway enrichment analysis of PDZK1IP1 related proteins

To explore the functional network of PDZK1IP1, we searched PDZK1IP1 related proteins on the String website and found that PDZK1IP1 may interact with ten proteins, including PDZK1, DMBT1, LCN2, IER5, SLC26A6, MPDZ, STIL, MTM1, OR51Q1, and TWIST1 (Fig. 4A). Then we carried out GO and KEGG pathway enrichment analysis by combining ten interacting protein genes on the String website and 100 related genes of PDZK1IP1 in the GEPIA database. Results are shown in Fig. 4B. We found it pertaining to MHC class II protein complex binding, neutrophil activation, neutrophil activation involved in immune response, and neutrophil degranulation. The above results showed that PDZK1IP1 might play a role in immune response in TME, especially with neutrophil activation.

### High expression of PDZK1IP1 in THCA

**Fig. 5:**
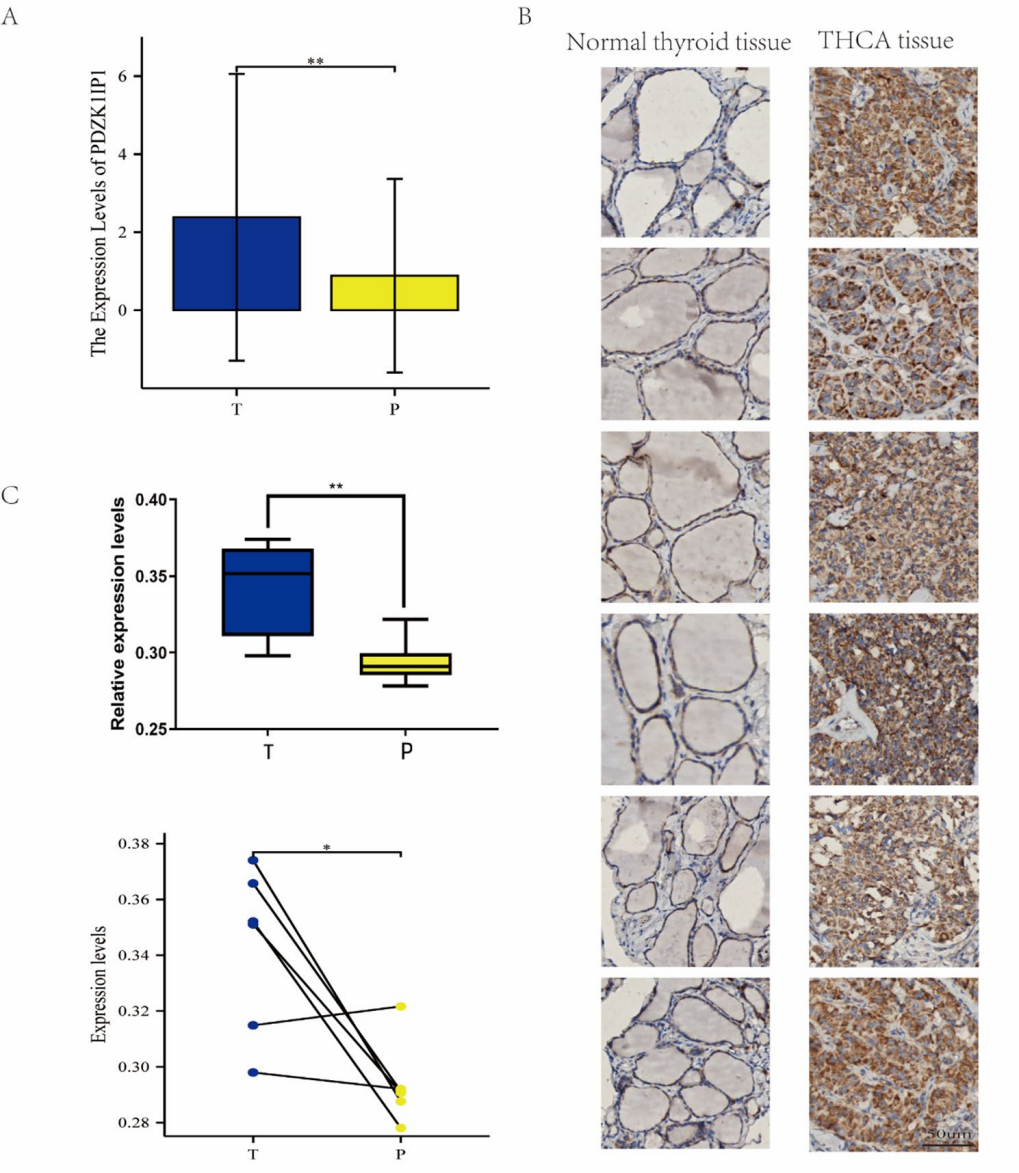
Results of RT-PCR and IHC in paired THCA and normal tissues. **A** Relative mRNA expression of PDZK1IP1 in 18 paired THCA and matched adjacent normal tissues, as determined by RT-PCR. **B** The IHC images of PDZK1IP1 protein expression in 6 fresh pairs of THCA and matched normal tissues. **C** The statistical analysis of IHC results of both overall and paired PDZK1IP1 expression levels respectively. T: tumor tissue; P: paracancerous tissue. *p < 0.05; **p < 0.01; ***p < 0.001

To experimentally validate our bioinformatic findings, we first assessed PDZK1IP1 expression in a cohort of 18 paired THCA and adjacent normal tissues. Quantitative RT-PCR analysis confirmed that the mRNA expression level of PDZK1IP1 in tumor tissue was significantly upregulated in tumor tissues compared to their matched adjacent normal tissues (*p* < 0.05) (Fig. 5A). Consistent with the mRNA data, the IHC results also showed that PDZK1IP1 was highly expressed in tumor tissues (Fig. 5B-C).

### Gene Set Enrichment Analysis (GSEA) of PDZK1IP1 in THCA

To further elucidate the biological functions associated with PDZK1IP1, we performed GSEA in the THCA cohort. The analysis revealed that genes positively correlated with PDZK1IP1 expression were significantly enriched in several immune-related pathways. Among the most significant enriched pathways were CD22 Mediated BCR Regulation, Antigen Activates B Cell Receptor and BCR Leading to Generation of Second (Supplementary Fig. 6). The results, derived from a rank-based method, strongly corroborated our initial GO and KEGG pathway enrichment analysis findings and further supported a critical role for PDZK1IP1 in modulating the immune and inflammatory landscape within the TME.

## Discussion

In this study, we conducted a comprehensive pan-cancer analysis to elucidate the role of PDZK1IP1 in tumorigenesis and tumor immunity. Our findings consistently demonstrated that PDZK1IP1 was frequently overexpressed at both the mRNA and protein levels in a wide range of cancers, including THCA, CHOL, COAD, LUAD, and PAAD, when compared to adjacent normal tissues. Furthermore, we found that its expression positively correlated with advanced tumor stage in several malignancies. It was similar to other studies [31–33]. A diagnostic value of PDZK1IP1 was also achieved in KICH, THCA, READ, and COAD. Our prognostic analysis revealed a context-dependent role for PDZK1IP1, where its overexpression was associated with either poor or favorable survival outcomes depending on the cancer type, a dichotomy also observed in prior research [29]. At the same time, its high expression was associated with poor prognosis in THCA, CESC, and THYM. These suggested that PDZK1IP1 could be used as a prognostic indicator in some tumors. The above results could be speculated that PDZK1IP1 can act as a tumor marker in a variety of cancers and may participate in tumor progression.

A key contribution of our study is the systematic investigation of PDZK1IP1’s role within TME. In tumor development and progression, the interaction between tumor and immune system, especially the mechanism of maintaining immunological self-tolerance and hindering effective tumor immunity, is critical. In the early stage of tumorigenesis, tumor cells are initially eliminated by the immune system, then tumor variant generates, and then immune escape occurs. These variants are amplified and appear tumor progression [34, 35]. Therefore, the interaction between immune cell infiltration and tumor in TME is essential for evaluating the effect of tumor immunotherapy and can be used as a prognosis indicator [36]. We demonstrated a broad association between PDZK1IP1 and the tumor immune landscape. For instance, PDZK1IP1 expression was significantly positively correlated with ICP genes in 16 tumors. This was particularly evident in THCA, where PDZK1IP1 expression was associated with 34 ICP genes. This finding was further supported by our analysis of immune cell infiltration, which showed a significant correlation between PDZK1IP1 expression and the abundance of immune infiltrates in 32 tumor types. Again, this association was especially pronounced in THCA, where PDZK1IP1 expression was linked to the infiltration of five kinds of immune cells: B cells, CD4 + T cells, CD8 + T cells, neutrophils, and dendritic cells. Moreover, we found that PDZK1IP1 expression was significantly correlated with 25 tumors in ESTIMATE score and a variety of tumors in TMB and Neoantigen. Taken together, these data strongly suggested that PDZK1IP1 may regulate tumor immunity by participating in the immune mechanism in TME.

With an in-depth study of the tumor, tumor stemness has attracted increasing attention. There is growing evidence that tumor stemness is related to invasive behavior, including tumor spread and metastasis. The latest research showed that the induction of tumor stemness in non-stem cancer cells can lead to treatment resistance and recurrence [37, 38]. Our study found that the expression of PDZK1IP1 was significantly correlated in 19 tumors. The positive correlation observed in eight of these tumors, including our focus cancer THCA, provided a potential mechanistic explanation for its association with patient prognosis.

Our pan-cancer analysis revealed that PDZK1IP1 is a significant prognostic and immunological marker across various malignancies. Given the clinical challenges and the emerging role of immunotherapy in THCA, as detailed in the Background, our experimental validation in THCA samples confirms that PDZK1IP1 is a robust oncogenic candidate in this specific disease setting.

While our study primarily delineates the clinical and immunological correlations of PDZK1IP1 across cancers, a deeper look into its established molecular functions provides a mechanistic basis for our observations. The primary mechanism of PDZK1IP1 appears to be its function as a scaffolding protein. Its C-terminal encoding the PDZ domain enables it to interact with PDZ domain-containing proteins such as PDZK1 and NHERF family members, thereby organizing and localizing signaling complexes at the plasma membrane [2, 3]. This scaffolding function is likely central to how it influences the diverse pathways highlighted in our analysis, including the immune response.

Several specific pathways have been identified. Notably, PDZK1IP1 is a potent inducer of ROS [8]. Elevated ROS levels contribute to genomic instability, promote cell proliferation, and create a pro-inflammatory microenvironment, which aligns with our findings of PDZK1IP1’s association with tumorigenesis and poor prognosis. Furthermore, a critical mechanism linking PDZK1IP1 to tumor progression is its activation of the Notch signaling pathway. It achieves this by directly binding to and sequestering NUMB, a negative regulator of Notch [9]. This action unleashes Notch signaling, a key driver of cancer stem cell maintenance and self-renewal. This provides a direct biological explanation for our observation that PDZK1IP1 expression positively correlates with the tumor stemness index in several cancers, including THCA. Additionally, PDZK1IP1 has been shown to modulate other critical cancer-related pathways, such as inhibiting TGF-β signaling by trapping Smad4 [11].

Therefore, PDZK1IP1 likely exerts its effects not through a single enzymatic activity but as a multifaceted signaling hub. Its ability to organize protein complexes, elevate oxidative stress, and activate oncogenic pathways like Notch collectively provides a strong mechanistic framework for understanding its role in promoting tumor progression, shaping the immune microenvironment, and serving as a robust prognostic biomarker.

Our PPI network analysis provides further mechanistic insight into the potential functions of PDZK1IP1 within the TME. The identification of ten potential interacting partners suggests that PDZK1IP1 likely functions as part of a larger protein complex rather than in isolation. Among these interactors, several proteins have well-established roles that strongly support our findings from the functional enrichment analysis.

Notably, the interaction with LCN2 and DMBT1 is particularly compelling. LCN2 is a crucial component of the innate immune system, known for its role in modulating inflammation and regulating neutrophil function, including its degranulation and chemotaxis [39]. Similarly, DMBT1 is recognized as a pattern recognition receptor involved in innate immunity and inflammatory responses [40]. The direct interaction with these proteins provides a strong mechanistic basis for our GO analysis results, which highlighted pathways such as neutrophil activation, neutrophil degranulation, and immune response. This suggests that PDZK1IP1 may directly participate in regulating neutrophil-mediated immunity by scaffolding or stabilizing these key immune-related proteins.

Furthermore, the network includes proteins linked to cancer progression and cell signaling. For instance, TWIST1 is a master regulator of epithelial-mesenchymal transition, a critical process for tumor invasion and metastasis [41]. The interaction with scaffolding proteins like PDZK1 and MPDZ, both containing PDZ domains, suggests that PDZK1IP1 may serve as a hub for organizing signaling complexes that influence not only immune responses but also tumor cell adhesion and migration [42, 43]. Other interactors such as STIL and IER5 further link PDZK1IP1 to fundamental cellular processes often dysregulated in cancer [44, 45].

Collectively, this network of interactions paints a picture of PDZK1IP1 as a multifaceted protein. Its connections to key innate immunity regulators (LCN2, DMBT1) and central cancer progression factors (TWIST1, STIL) strongly support our hypothesis that PDZK1IP1 plays a pivotal role in the TME, potentially by bridging the gap between tumor cell-intrinsic malignant pathways and the extrinsic immune response. Meanwhile, our analysis of the pathological tissues of THCA showed that PDZK1IP1 was highly expressed in both mRNA and protein levels compared with the adjacent tissues, and it was consistent with the other previous researches [46, 47], indicating that PDZK1IP1 was an oncogene in THCA and promoted the development of THCA. ALL these findings above showed that PDZK1IP1 could be used as a marker and target of tumor immunotherapy in THCA, which provided the basis for further exploration of new treatment methods.

As a nonglycosylated membrane protein that is significantly overexpressed in malignant tissues compared to normal tissues, PDZK1IP1 represents a viable target for antibody-based therapies, such as monoclonal antibodies or antibody-drug conjugates. Furthermore, our findings demonstrated a strong correlation between PDZK1IP1 expression and ICP genes and TMB, which suggests that targeting it could potentially promote anti-tumor immunity. Additionally, given the association we identified between PDZK1IP1 and neutrophil activation pathways, therapeutic modulation of this protein might alter neutrophil-mediated inflammatory responses, potentially shifting the TME from a pro-tumorigenic to an anti-tumorigenic state. While these applications are theoretical, our results provide a strong rationale for future studies to explore PDZK1IP1-targeted interventions in combination with standard immunotherapies, particularly in THCA.

The research paradigm employed in our study, which transitions from a broad pan-cancer bioinformatic screen to focused experimental validation in a specific cancer type, is a robust and increasingly common approach for identifying novel cancer biomarkers. This methodology is exemplified by several recent studies recommended for discussion. For instance, comprehensive analyses of Bystin [48] and CENPN [49] followed a similar trajectory, revealing their roles as prognostic and immune biomarkers first through pan-cancer analysis and subsequently validating these findings in breast cancer. Our results for PDZK1IP1 align closely with the patterns observed in these studies, as well as with the findings for DBNDD1 in breast cancer [50], where overexpression of the target gene consistently correlates with poor prognosis and modulation of the tumor immune microenvironment.

While these studies collectively establish a recurring theme of identifying immune-related oncogenes, our work provides a distinct and novel contribution. Firstly, we introduce PDZK1IP1 as a new and previously under-characterized player in this context. Secondly, our validation in THCA extends this research paradigm to a different, endocrine-related malignancy, offering unique insights. Our identification of PDZK1IP1’s strong association with immune checkpoints and TME components in THCA suggests its potential as a valuable biomarker for predicting prognosis and guiding immunotherapy strategies in this specific disease setting. Therefore, our study not only reinforces a successful discovery model but also expands the landscape of actionable immune-related targets to include PDZK1IP1 in THCA.

This study has several limitations that should be acknowledged. A primary limitation is that our findings are largely based on bioinformatic analyses coupled with preliminary validation in clinical samples. We have not performed in-depth functional experiments at the cellular or animal levels. Therefore, while our results strongly suggest a role for PDZK1IP1 in tumor immunity, the precise molecular mechanisms driving these associations remain to be elucidated. Future studies, including in vitro experiments using cell lines with PDZK1IP1 knockdown or overexpression, as well as in vivo studies using animal models, are essential to causally link PDZK1IP1 expression to tumor growth, metastasis, and the immune microenvironment dynamics we observed. Secondly, the bioinformatic analysis itself has inherent constraints. We observed some inconsistencies in PDZK1IP1 expression levels for certain tumors across different public databases, which may introduce a degree of deviation into our pan-cancer conclusions. Thirdly, regarding our experimental validation in THCA, the sample size was relatively small. Although our results were statistically significant, a larger cohort is necessary to more robustly confirm the bioinformatic predictions. Furthermore, this validation cohort was not analyzed for patient prognosis, meaning the impact of PDZK1IP1 on clinical outcomes in our own patient group remains to be verified. Larger, prospective clinical studies are therefore needed to firmly establish its utility as a prognostic and predictive biomarker in THCA. Finally, while our analysis connects PDZK1IP1 to tumor immunity, particularly in THCA, the specific mechanisms are still largely unknown and require further experimental verification.

## Conclusions

Our study inferred that PDZK1IP1 was not only an oncogene, which was related to the occurrence and development of tumors but also related to tumor immunity. It could be used as a potential target of antitumor immunotherapy, especially for THCA.

## Supplementary Information

Supplementary Material 1. Supplementary Figures 1-6 are available in the Supplementary information for comprehensive image analysis.

## Data Availability

The data could be obtained by contacting the corresponding author.

## Abbreviations

PDZK1IP1: PDZK1IP1-interacting protein 1
ROS: Reactive oxygen species
OS: Overall survival
RFS: Relapse-free survival
HRs: Hazard ratios
TMB: Tumor mutational burden
ICP: Immune checkpoint
TME: Tumor microenvironment
DMPsi: Differentially methylated probes-based stemness index
GO: Gene ontology
KEGG: Kyoto encyclopedia of genes and genomes
PTMC: Papillary thyroid carcinoma
MTC: Medullary carcinoma
RT-PCR: Real-time polymerase chain reaction
CHOL: Cholangiocarcinoma
COAD: Colon adenocarcinoma
LIHC: Liver hepatocellular carcinoma
LUAD: Lung adenocarcinoma
READ: Rectum adenocarcinoma
UCEC: Uterine corpus endometrial carcinoma
KICH: Kidney chromophobe
PAAD: Pancreatic adenocarcinoma
UCEC: Uterine corpus endometrial carcinoma
BRCA: Breast invasive carcinoma
CESC: Cervical squamous cell carcinoma and endocervical Adenocarcinoma
LUSC: Ung squamous cell carcinoma
THYM: Thymoma
HNSC: Head and neck squamous cell carcinoma
KIRP: Kidney renal papillary cell carcinoma
ESCA: Esophageal carcinoma
STAD: Stomach adenocarcinoma
ICP: Immune checkpoint
MESO: Mesothelioma
UVM: Uveal melanoma
