# Supplementary material for: Pan-cancer analysis of PDZK1IP1 reveals its role in tumorigenesis and tumor immunity: focused validation in thyroid carcinoma
Source: Hereditas. 2026 Feb 27;163:45. doi: 10.1186/s41065-026-00662-1 (PMC13049858; doi:10.1186/s41065-026-00662-1)
Supplement: Supplementary file 1 — Supplementary Material 1. Supplementary Figures 1-6 are available in the Supplementary information for comprehensive image analysis. [file 41065_2026_662_MOESM1_ESM.docx]

**Supplementary Information**

**
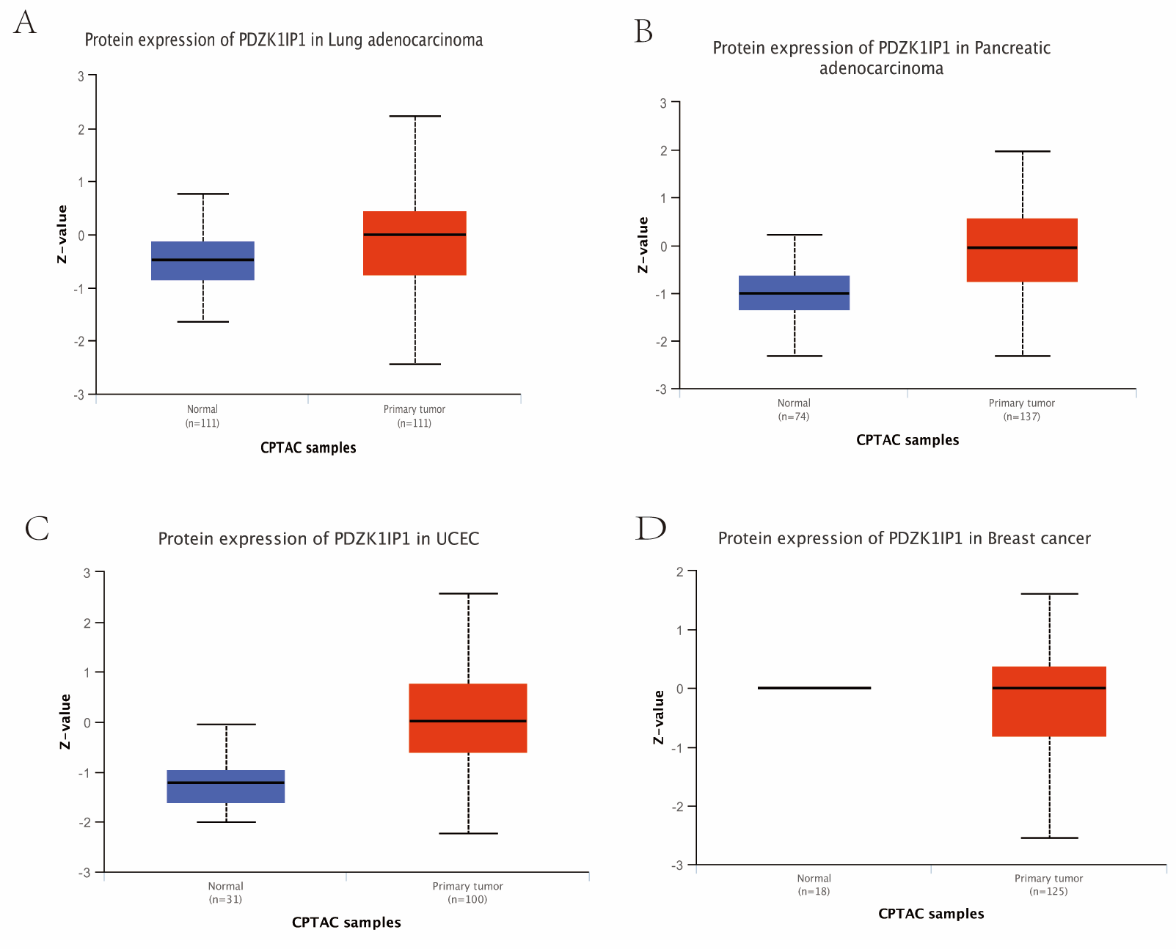
**

**Supplementary Fig. 1** PDZK1IP1 expression in differential cancers in CPTAC database.
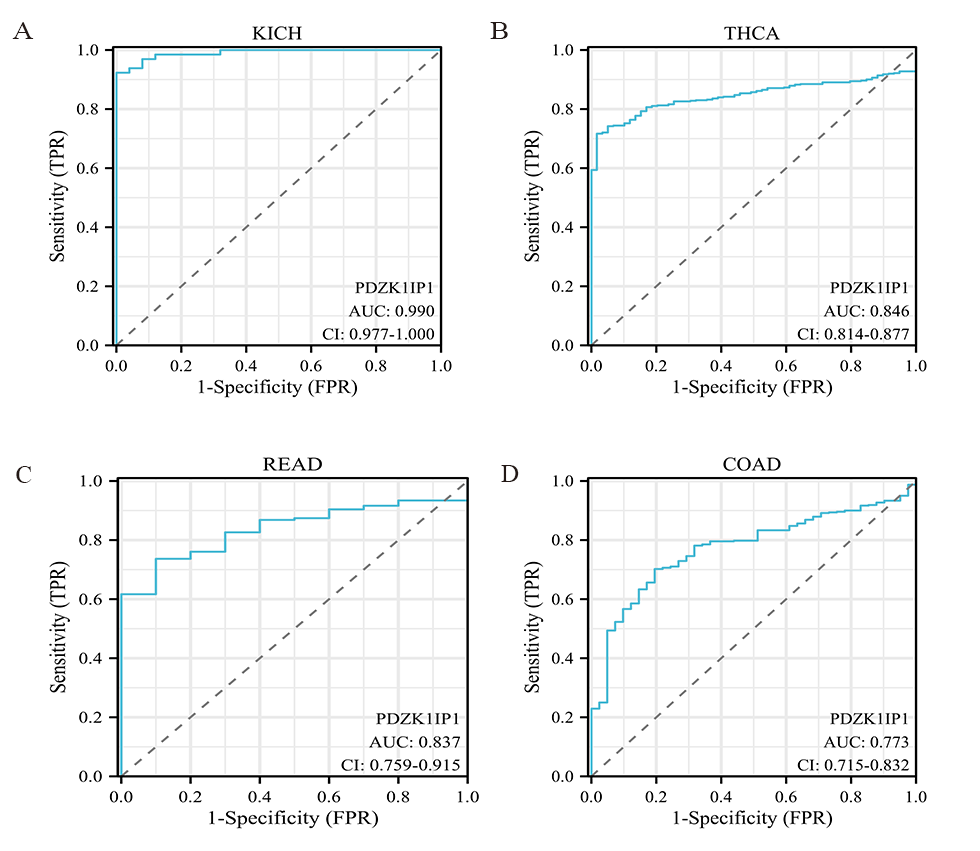


**Supplementary Fig.2** The diagnostic value of PDZK1IP1 expression in distinguishing tumor tissues from adjacent normal tissues.

**
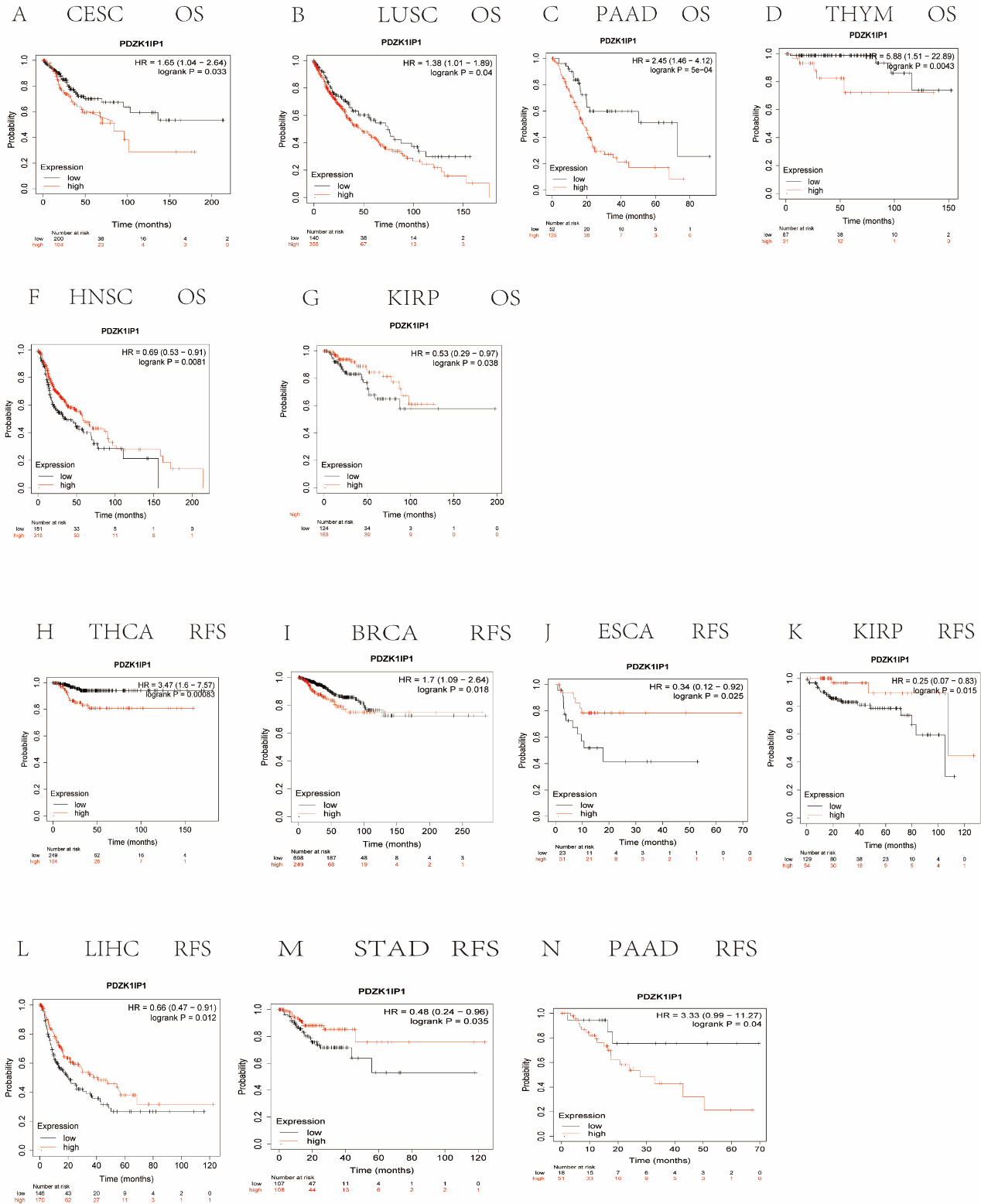
**

**Supplementary Fig.3** Kaplan-Meier survival curve of human cancers with high and low PDZK1IP1 expression in differential cancers in the Kaplan Meier plotter database. (**A-D**)High expression of PDZK1IP1 was associated with poor OS in PAAD（HR=2.45, p=0.0005）, CESC (HR=1.65, *P*=0.033), LUSC (HR=1.38, p=0.04), and THYM (HR=5.88, *P*=0.0043). (**F-G**) High expression indicated better OS in HNSC (HR=0.69, p=0.0081) and KIRP (HR=0.53, p=0.038). (**H,N**) High expression of PDZK1IP1 was associated with poor RFS in THCA (HR = 3.47, p= 0.00083) and PAAD（HR=3.33, p=0.04）. (**I-M**) High expression indicated better RFS in BRCA (HR = 1.7, p= 0.018), ESCA (HR = 0.34, p= 0.025), KIRP (HR = 0.25, p= 0.015), LIHC (HR = 0.66, p= 0.012), and STAD (HR = 0.48, p= 0.035). OS, overall survival; RFS, relapse-free survival

**
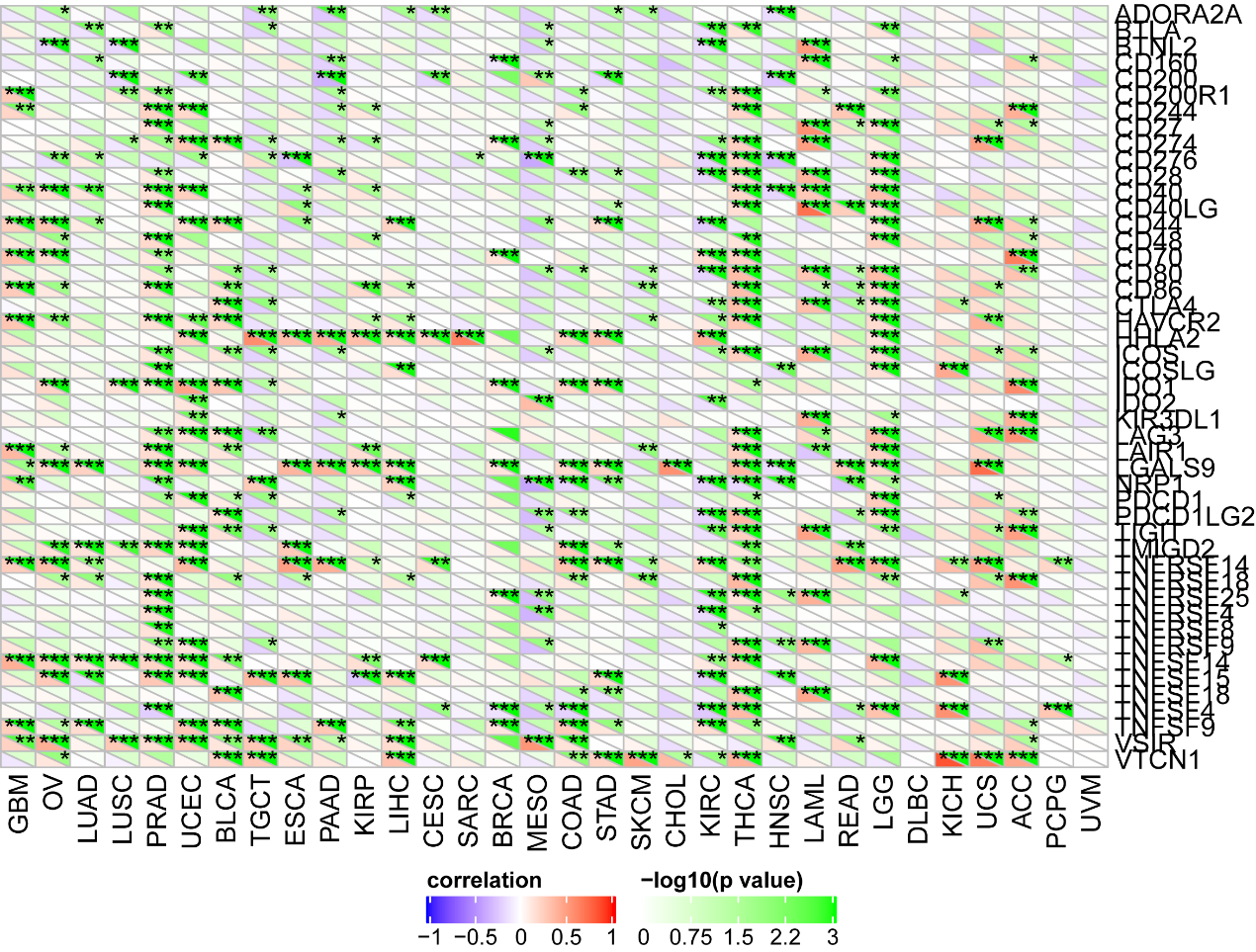
**

**Supplementary Fig. 4** The relationship between PDZK1IP1 expression and pan-cancer immune checkpoint genes. *p< 0.05; **p< 0.01; ***p< 0.001

**
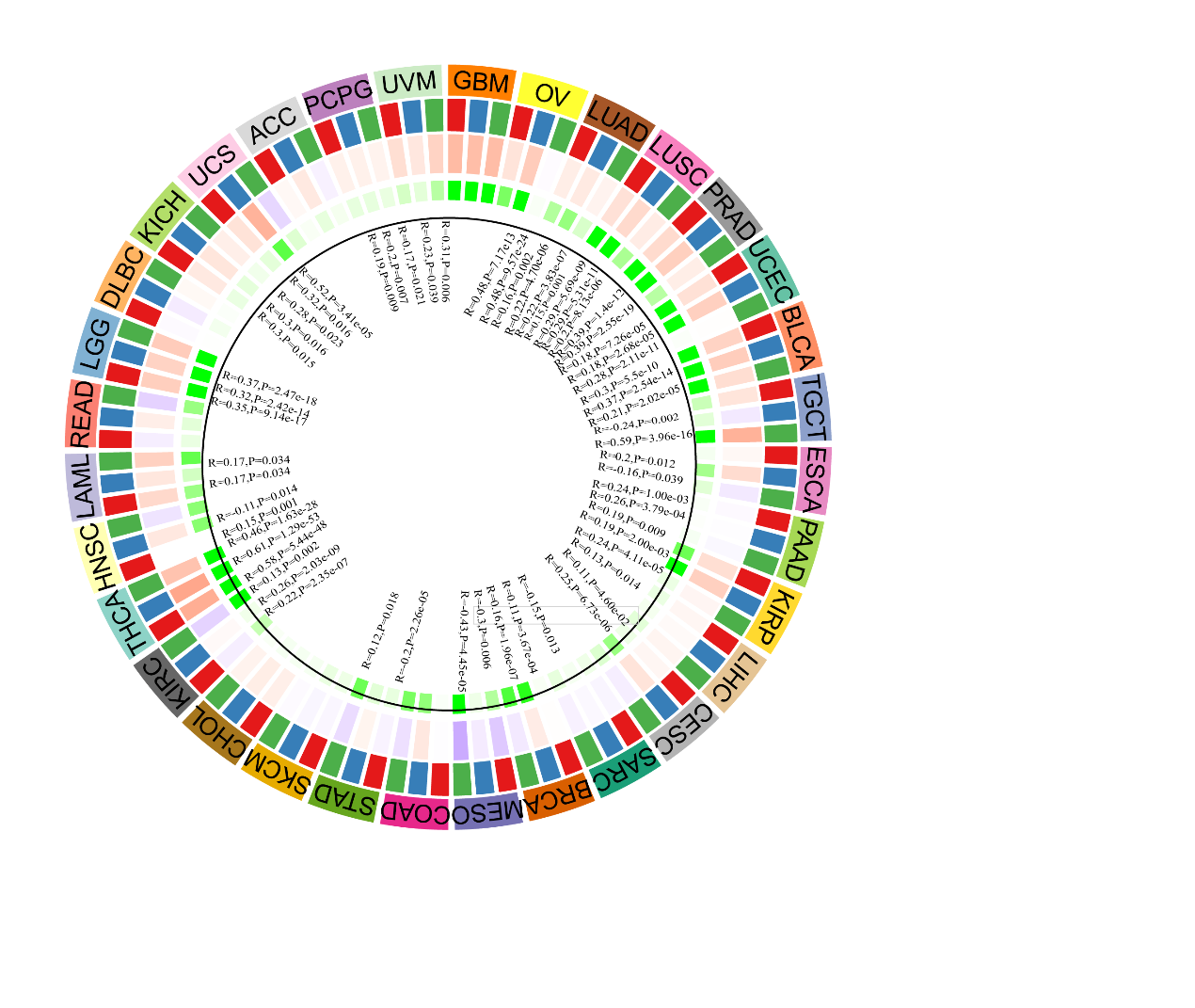
**

**Supplementary Fig. 5** The relationship between PDZK1IP1 expression and the ESTIMATE score in multiple cancers.


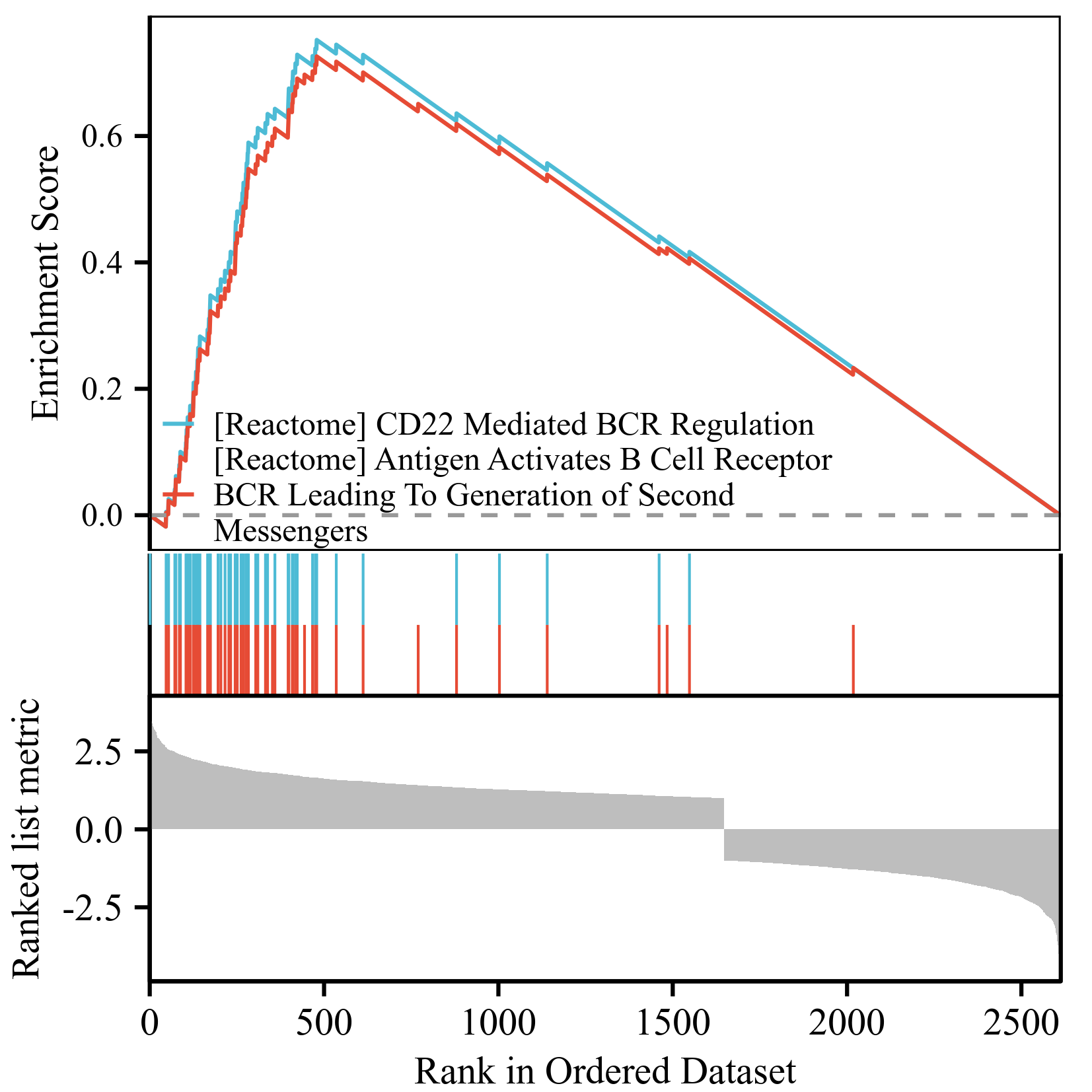


**Supplementary Fig. 6** Gene Set Enrichment Analysis (GSEA) of pathways associated with PDZK1IP1 expression in the TCGA-THCA cohort.
